# Neutrophil‐to‐Lymphocyte Ratio as a Prognostic Factor of Survival Outcomes in Head and Neck Squamous Cell Carcinoma Receiving Neoadjuvant Immunotherapy

**DOI:** 10.1002/cam4.71693

**Published:** 2026-03-22

**Authors:** Pablo Llerena, Praneet C. Kaki, Kathryn Nunes, Eric Mastrolonardo, Kelly Bridgham, Annie E. Moroco, Hani Samarah, Mouadh Barbirou, David Baek, Chun Wang, Voichita Bar Ad, David M. Cognetti, Joseph M. Curry, Jennifer M. Johnson, Hushan Yang, Larry A. Harshyne, Adam J. Luginbuhl

**Affiliations:** ^1^ Department of Otolaryngology—Head and Neck Surgery Thomas Jefferson University Hospital Philadelphia Pennsylvania USA; ^2^ Department of Medical Oncology Thomas Jefferson University Hospital Philadelphia Pennsylvania USA; ^3^ Sidney Kimmel Comprehensive Cancer Center Thomas Jefferson University Philadelphia Pennsylvania USA; ^4^ Sidney Kimmel Medical College Thomas Jefferson University Philadelphia Pennsylvania USA; ^5^ Department of Radiation Oncology Thomas Jefferson University Philadelphia Pennsylvania USA; ^6^ Department of Microbiology & Immunology Thomas Jefferson University Philadelphia Pennsylvania USA

**Keywords:** disease‐free survival, HNSCC, immune checkpoint inhibition, overall survival, serological markers

## Abstract

**Purpose:**

The neutrophil‐to‐lymphocyte ratio (NLR) is a prognostic marker in cancers treated with immune checkpoint inhibitors (ICI), reflecting the link between inflammation and cancer immune response. This study examines NLR's prognostic value in head and neck squamous cell carcinoma (HNSCC) patients receiving neoadjuvant ICI therapy, focusing on its potential as an independent predictor of overall survival (OS) and disease‐free survival (DFS).

**Methods:**

We conducted a retrospective cohort study including three neoadjuvant trials: durvalumab ± metformin, nivolumab ± tadalafil, or nivolumab ± BMS‐986205 from 2017 to 2022. Pre‐treatment NLR was calculated using absolute neutrophil and absolute lymphocyte counts obtained before neoadjuvant ICI initiation. The optimal pre‐treatment NLR cut‐off was identified using receiver operating characteristic (ROC) curve analysis. OS and DFS were assessed using Kaplan–Meier and multivariable Cox proportional hazards regression models.

**Results:**

A total of 97 patients met inclusion criteria. NLR < 4.14 was associated with improved overall survival (HR 0.07, 95% CI 0.01–0.30, *p* < 0.001) and DFS (HR 0.21, 95% CI 0.08–0.54, *p* = 0.001) compared to NLR ≥ 4.14. NLR < 4.14 remained independently associated with improved OS (HR 0.14, 95% CI 0.02–0.78, *p* = 0.025) and DFS (HR 0.25, 95% CI 0.07–0.87, *p* = 0.030) on multivariable Cox regression. The survival benefit of NLR < 4.14 persisted after sub‐stratification for p16 status, ICI pathologic response status, and ICI trial.

**Conclusion:**

Low NLR was independently associated with improved OS and DFS among patients with HNSCC who received neoadjuvant ICI. These findings suggest the potential utility of the NLR in improving patient selection.

## 1 Introduction

Management of locoregionally advanced head and neck squamous cell carcinoma (HNSCC) remains challenging with high rates of treatment failure and disease recurrence [1]. Recently, immune checkpoint inhibitors (ICIs) have been incorporated into the standard‐of‐care management of recurrent or metastatic (R/M) HNSCC. Pembrolizumab and nivolumab (anti‐programmed cell death 1 [PD‐1] antibodies) have demonstrated clinical benefits in R/M HNSCC compared to standard therapies [2, 3]. Despite encouraging results with ICIs in HNSCC, only 20%–30% of patients with R/M cancer are disease‐free at 2 years [2, 4]. To improve ICI response rates, alternative strategies, including the use of neoadjuvant ICI (nICI) before definitive treatment, are under exploration.

Although ICIs have demonstrated promise in select patients, treatment response varies significantly, highlighting the need for biomarkers that may predict clinical response. Previous studies have linked PD‐L1 expression and tumor mutational burden (TMB) with clinical response to ICI across several cancer types [5, 6]. However, PD‐L1 and TMB are limited by their requirement for sufficient tumor tissue and DNA sequencing, a lack of standardized immunohistochemistry scoring, and extensive financial costs [5, 7]. ICIs require thoughtful selection as they are associated with both a higher financial cost and risk of immune‐related adverse events (irAEs), but lower toxicity than traditional cytotoxic therapy [8]. Thus, there is a demand for low‐cost, readily accessible biomarkers with prognostic capabilities. Peripheral blood biomarkers such as the neutrophil‐lymphocyte ratio (NLR) may fulfill this role. High peripheral neutrophils and low lymphocytes reflect increased systemic and local inflammation that promotes an immune‐suppressive tumor microenvironment [9, 10, 11, 12]. NLR has been previously associated with survival outcomes in various solid tumors, including in patients with HNSCC [11, 13]. It is unknown whether NLR is associated with improved survival after ICI treatment for HNSCC in the neoadjuvant setting [8]. The purpose of this study is to investigate the prognostic utility of pre‐treatment NLR in patients that received nICI for HNSCC.

## 2 Methods

### 2.1 Study Population and Design

Patients from three multi‐institutional window‐of‐opportunity clinical trials between May 2017 and January 2022 were included in this pooled analysis. The clinical trials included treatment with durvalumab, an anti‐programmed death‐ligand 1 (aPDL1) ICI, with or without metformin (Durva±Met; NCT03618654); treatment with an anti‐programmed death 1 (aPD1) nivolumab with or without an IDO1 inhibitor (BMS986205) (Nivo±IDO; NCT03854032) and treatment with nivolumab with or without tadalafil (Nivo±Tad; NCT03238365). Specific details regarding inclusion criteria and methodology are found in Supporting Information [14, 15, 16]. All clinical trials were conducted with approval by the Thomas Jefferson University Institutional Review Board (IRB #21E.431) with written informed consent from each participant.

### 2.2 Outcome Measures

Demographic, clinical, and pathologic data including age, sex, race, body mass index (BMI), smoking status, primary disease site, p16 status, pathologic AJCC tumor staging, and adjuvant therapy administration were collected. Immune‐related adverse events (irAEs) of any grade were reported. Baseline complete blood count with differential to quantify neutrophils and lymphocytes was collected 1 week prior to nICI treatment. NLR was calculated by dividing the absolute neutrophil count by the absolute lymphocyte count. The primary outcomes were overall survival (OS) and disease‐free survival (DFS), which were assessed at the most recent follow‐up and calculated from completion of all treatment. Treatment response was defined as a pathologic treatment effect (pTE) > 20%, as previously published in the Nivo±Tad trial while the Durva±Met trial utilized a pTE > 10% [16].

### 2.3 Peripheral Cytokine Analysis

Peripheral whole blood samples were taken at the time of study recruitment (pre‐treatment), between completion of the neoadjuvant investigational agents (on treatment), and prior to definitive surgical resection (post‐treatment). Detailed cytokine methodology has previously been published by Alnemri et al. [17].

### 2.4 Statistical Analysis

Descriptive statistics were used to characterize the study population and report demographic, clinical, and pathologic characteristics. Continuous variables were reported as mean (standard deviation, [SD]) and analyzed using paired, nonparametric Wilcoxon Rank Sum Testing. Categorical variables were reported as frequency (percentage) and analyzed using chi‐square (*χ* ^2^) tests. To determine the NLR cutoff, we performed receiver operating characteristic (ROC) curve analysis using overall survival (OS; AUC = 83.2%) and disease‐free survival (DFS; AUC = 70.3%) as endpoints. The optimal threshold was defined by the Youden index (maximizing sensitivity and specificity), yielding an NLR cut point of 4.14. Patients were therefore categorized into High‐NLR (≥ 4.14) and Low‐NLR (< 4.14) groups. Other continuous variables (including BMI, neutrophil count, and lymphocyte count) were dichotomized according to their respective cohort medians. Pre‐ICI treatment, post‐ICI treatment, and changes (post‐ICI—pre‐ICI) in peripheral cytokines were compared between H‐NLR and L‐NLR cohorts. Kaplan–Meier survival analysis and log‐rank testing were implemented to compare OS and DFS between the two cohorts at the time of most recent follow‐up. A Cox proportional hazards regression model handling missing data with listwise elimination and adjusting for variables significant on univariable regression models was used to identify independent predictors of OS and DFS.

To present a comparative cohort, we identified a matched control cohort of HNSCC patients who underwent surgery without neoadjuvant ICI. Exclusion criteria matched that of the experimental cohort, with all subjects completing NCCN adjuvant guideline therapy post operatively. Kaplan–Meier survival and univariable Cox regression analyses were performed to compare OS and DFS in patients with H‐NLR and L‐NLR in this control cohort to determine its specificity, or lack thereof, for neoadjuvant ICI treatment of HNSCC. Significance was set to an *α* level = 0.05 for all tests. All statistical analyses were performed using R Studio Version 2023.03.1.

## 3 Results

### 3.1 Patient and Clinical Trial Characteristics

Of 138 patients enrolled across the three clinical trials, 41 were excluded due to trial withdrawal or missing data, leaving a total of 97 patients included for analysis (Table 1).

**TABLE 1:** Patient and clinicopathologic characteristics by pre‐ICI NLR.

Characteristic	*N*	Overall, *N* = 97^a^	NLRpre‐H, *N* = 24^a^	NLRpre‐L, *N* = 73^a^	*p* ^b^
Age (years)	97	61.59 (10.62)	62.21 (9.16)	61.38 (11.11)	0.7
Sex	97				0.8
Female		16 (16%)	3 (12%)	13 (18%)	
Male		81 (84%)	21 (88%)	60 (82%)	
Race	97				0.5
Black or African American		6 (6.2%)	0 (0%)	6 (8.2%)	
Other Pacific Islander		1 (1.0%)	0 (0%)	1 (1.4%)	
White		90 (93%)	24 (100%)	66 (90%)	
Disease site	97				0.8
Hypopharynx		3 (3.1%)	1 (4.2%)	2 (2.7%)	
Larynx		4 (4.1%)	2 (8.3%)	2 (2.7%)	
Oral cavity		28 (29%)	7 (29%)	21 (29%)	
Oropharynx		59 (61%)	14 (58%)	45 (62%)	
Sinonasal		2 (2.1%)	0 (0%)	2 (2.7%)	
Unknown		1 (1.0%)	0 (0%)	1 (1.4%)	
Experienced recurrence following ICI treatment	97	14 (14%)	7 (29%)	7 (9.6%)	0.039
Baseline BMI (kg/m^2^)	96	28.86 (6.91)	26.30 (5.49)	29.66 (7.14)	0.023
Smoking status	97				0.016
Current		18 (19%)	5 (21%)	13 (18%)	
Former		40 (41%)	15 (62%)	25 (34%)	
Never		39 (40%)	4 (17%)	35 (48%)	
p16+ HPV‐related disease	97	56 (58%)	12 (50%)	44 (60%)	0.4
Pathologic AJCC stage	96				0.5
0		5 (5.2%)	0 (0%)	5 (6.8%)	
I		48 (50%)	9 (39%)	39 (53%)	
II		11 (11%)	4 (17%)	7 (9.6%)	
III		6 (6.2%)	2 (8.7%)	4 (5.5%)	
IVa		20 (21%)	6 (26%)	14 (19%)	
IVb		6 (6.2%)	2 (8.7%)	4 (5.5%)	
Follow‐up duration (months)	97	34.32 (15.94)	31.00 (20.42)	35.41 (14.17)	0.2

^a^ Mean (SD); *n* (%).

^b^ Wilcoxon rank sum test; Fisher's exact test; Pearson's Chi‐squared test.

The average pre‐treatment NLR across the cohort was 3.54 (SD 2.29). 73 patients were classified as Low‐NLR (L‐NLR) (average NLR 2.59, SD 0.79), and 24 patients were classified as High‐NLR (H‐NLR) (average NLR 6.42, SD 2.90). Patients with H‐NLR prior to receiving immunotherapy were more likely to experience disease recurrence compared to patients with L‐NLR (29% vs. 9.6%, *p* = 0.039) (Table 2). Pathological treatment response to ICI therapy was achieved in 42% of patients and did not differ between patients with H‐NLR and L‐NLR (46% vs. 41%, *p* = 0.700). irAEs were experienced by 26% of patients and occurred at similar rates among patients with H‐NLR and L‐NLR (25% vs. 26%, *p* > 0.9).

**TABLE 2:** Logistic Cox Regression Identifying Predictors of Overall Survival.

Variable	Univariate analysis			Multivariate analysis		
	HR	95% CI	*p*	HR	95% CI	*p*
Male	1.74	0.22–13.62	0.598	—	—	—
Median age	0.40	0.11–1.51	0.177	—	—	—
Yes smoker	2.89	0.62–13.46	0.176	—	—	—
< Median BMI	2.45	0.74–8.15	0.143	—	—	—
P16+	**0.25**	**0.067–0.97**	**0.04**	0.69	0.14–3.34	0.65
ICI adverse event	0.31	0.039–2.40	0.259	—	—	—
pT2/3/4	**7.86**	**1.00–61.63**	**0.05**	0.42	0.2–23.0	0.50
PN1‐3	**0.26**	**0.076–0.88**	**0.03**	—	—	—
AJCC III/IV	3.02	0.91–10.06	0.0711	—	—	—
Median neutrophils	**0.10**	**0.013–0.81**	**0.030**	0.34	0.03	3.25
Median lymphocytes	3.38	0.89–12.79	0.0728	—	—	—
Low pre ICI NLR	**0.0665**	**0.014–0.300**	**< 0.0001**	0.13	0.02	0.77

*Note:* Bold values indicate statistical significance (*p* < 0.05).

### 3.2 Pre‐ICI Neutrophil‐to‐Lymphocyte Ratio as a Predictor of Survival

**FIGURE 1:**
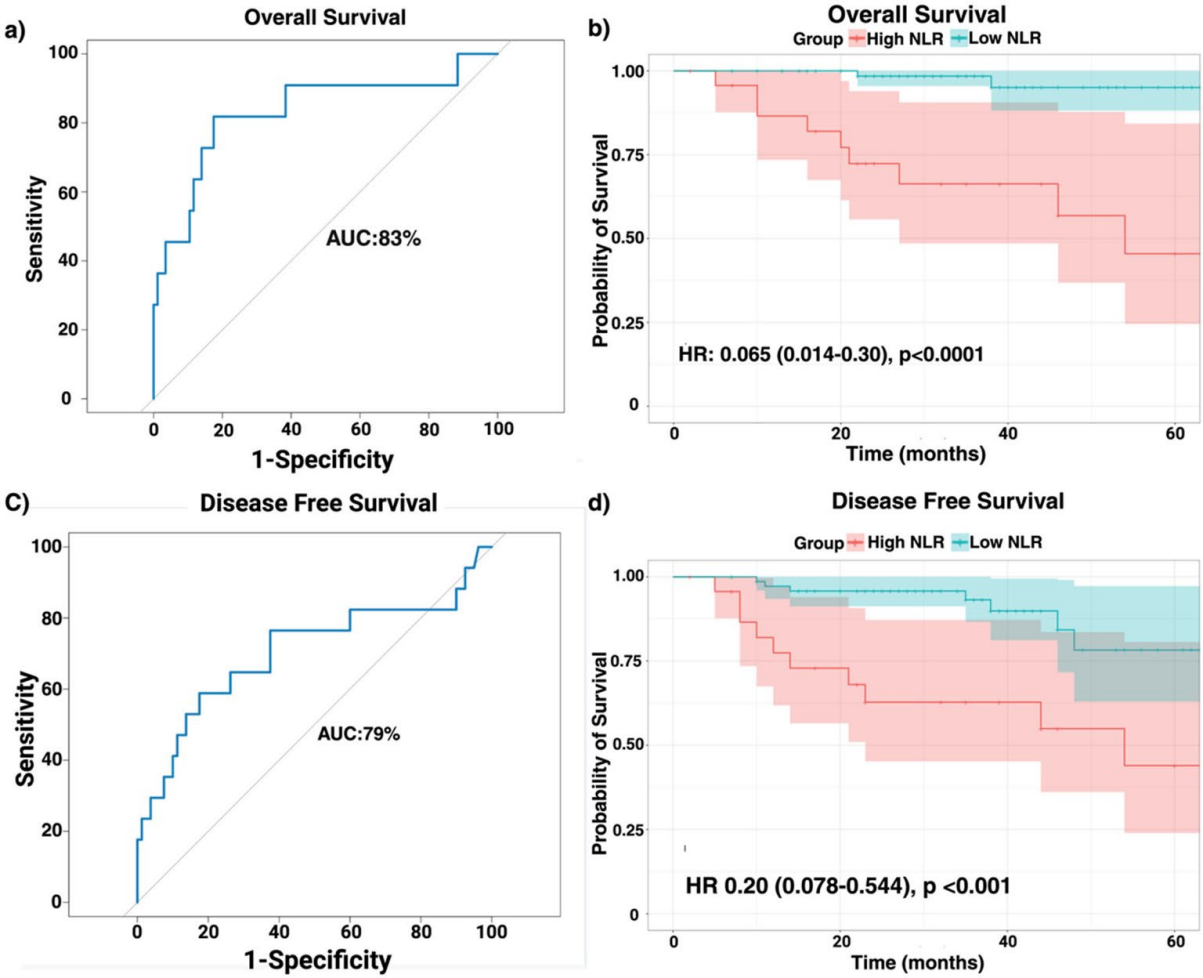
Impact of Neutrophil‐to‐Lymphocyte Ratio (NLR) on Survival Outcomes in HNSCC Patients Treated with Neoadjuvant ICI. (a) ROC curve demonstrating the discriminatory ability of NLR for predicting OS (AUC = 0.83). (b) Kaplan‐Meier Curve for OS: Patients with NLR < 4.14 show significantly improved OS (HR 0.065, 95% CI 0.014–0.30, *p* < 0.0001). (c) ROC curve demonstrating the discriminatory ability of NLR for predicting DFS (AUC = 0.79). (d) Kaplan‐Meier Curve for DFS: NLR < 4.14 is associated with improved DFS (HR 0.20, 95% CI 0.078–0.544, *p* < 0.001).

**FIGURE 2:**
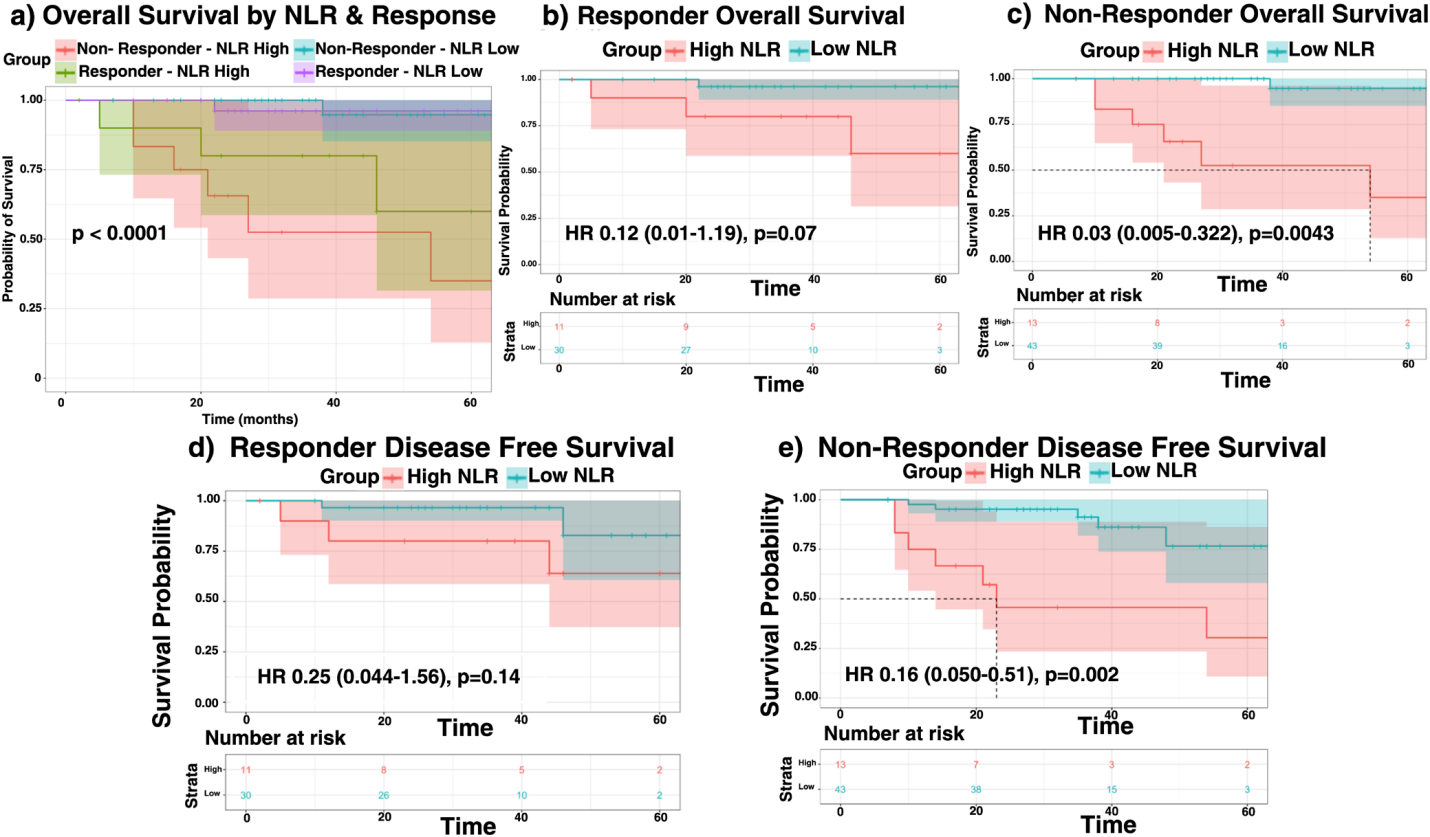
Kaplan–Meier survival analysis stratified by NLR and pathologic treatment effect (PTE). (a) Overall survival by NLR and response status: Significant differences in OS by NLR and PTE status (*p* < 0.0001). (b) Responder overall survival: Improved OS in low NLR responders, approaching significance (HR 0.12, 95% CI 0.01–1.19, *p* = 0.07). (c) Non‐responder overall survival: Low NLR non‐responders show significantly better OS (HR 0.03, 95% CI 0.005–0.322, *p* = 0.0043). (d) Responder disease‐free survival: No significant difference in DFS among responders by NLR (HR 0.25, 95% CI 0.044–1.56, *p* = 0.14). (e) Non‐responder disease‐free survival: Low NLR non‐responders exhibit significantly improved DFS (HR 0.16, 95% CI 0.050–0.51, *p* = 0.002).

The median time to most recent follow up for patients stratified by ICI trial are as follows: Nivo±Tad: 53 months (14–67), Nivo±IDO: 24 months (2–44), Durva±Met: 36 months (7–56). Kaplan–Meier survival demonstrated that patients with L‐NLR had improved OS (HR 0.07, 95% CI 0.01–0.30, *p* < 0.001) and DFS (HR 0.21, 95% CI 0.08–0.54, *p* = 0.001) compared to patients with H‐NLR (Figure 1). The observed survival benefit of pre‐treatment L‐NLR persisted for patients regardless of p16 status, type of nICI treatment, or category of pathologic treatment response (pTR) (Figure 2).

### 3.3 Univariable and Multivariable Analysis of Predictors of Overall (OS) and Disease‐Free Survival

Factors associated with improved OS on univariable analysis included L‐NLR (HR 0.07, 95% CI 0.01–0.30, *p* < 0.001), absolute neutrophil count (ANC) below the median of cohort, p16‐positivity, and pN1‐3 stage, and are summarized in Table 3. pT2+ staging was associated with a worse OS on univariable Cox regression. Upon multivariable Cox regression, only L‐NLR remained independently associated with improved OS (HR 0.14, 95% CI 0.02–0.78, *p* = 0.025).

**TABLE 3:** Logistic Cox Regression Identifying Predictors of Disease‐Free Survival.

Variable	Univariate analysis			Multivariate analysis		
	HR	95% CI	*p*	HR	95% CI	*p*
Male	0.55	0.18–1.68	0.291	—	—	—
Median age	0.60	0.22–1.62	0.311	—	—	—
History smoker	1.14	0.42–3.10	0.803	—	—	—
Median BMI	1.662	0.59–4.43	0.345	—	—	—
P16+	**0.20**	**0.066–0.623**	**0.004**	0.37	0.06–2.75	0.37
ICI adverse event	0.19	0.025–1.426	0.106	—	—	—
pT2/3/4	**3.60**	**1.03–12.54**	**0.05**	1.24	0.25–5.75	0.82
PN1‐3	**0.30**	**0.11–0.83**	**0.02**	0.51	0.14–1.86	0.31
AJCC III/IV	**3.57**	**1.35–9.45**	**0.01**	0.87	0.19–0.39	0.87
< Median neutrophils	**0.14**	**0.03–0.61**	**0.008**	0.36	0.07–1.835	0.03
< Median lymphocytes	1.83	0.69–4.83	0.223	—	—	—
Low pre ICI NLR	**0.21**	**0.078–0.54**	**0.001**	**0.21**	**0.078–0.54**	**0.001**

*Note:* Bold values indicate statistical significance (*p* < 0.05).

### 3.4 Assessing Neutrophil‐to‐Lymphocyte Ratio as a Prognostic Marker in a Control Patient Cohort

**FIGURE 3:**
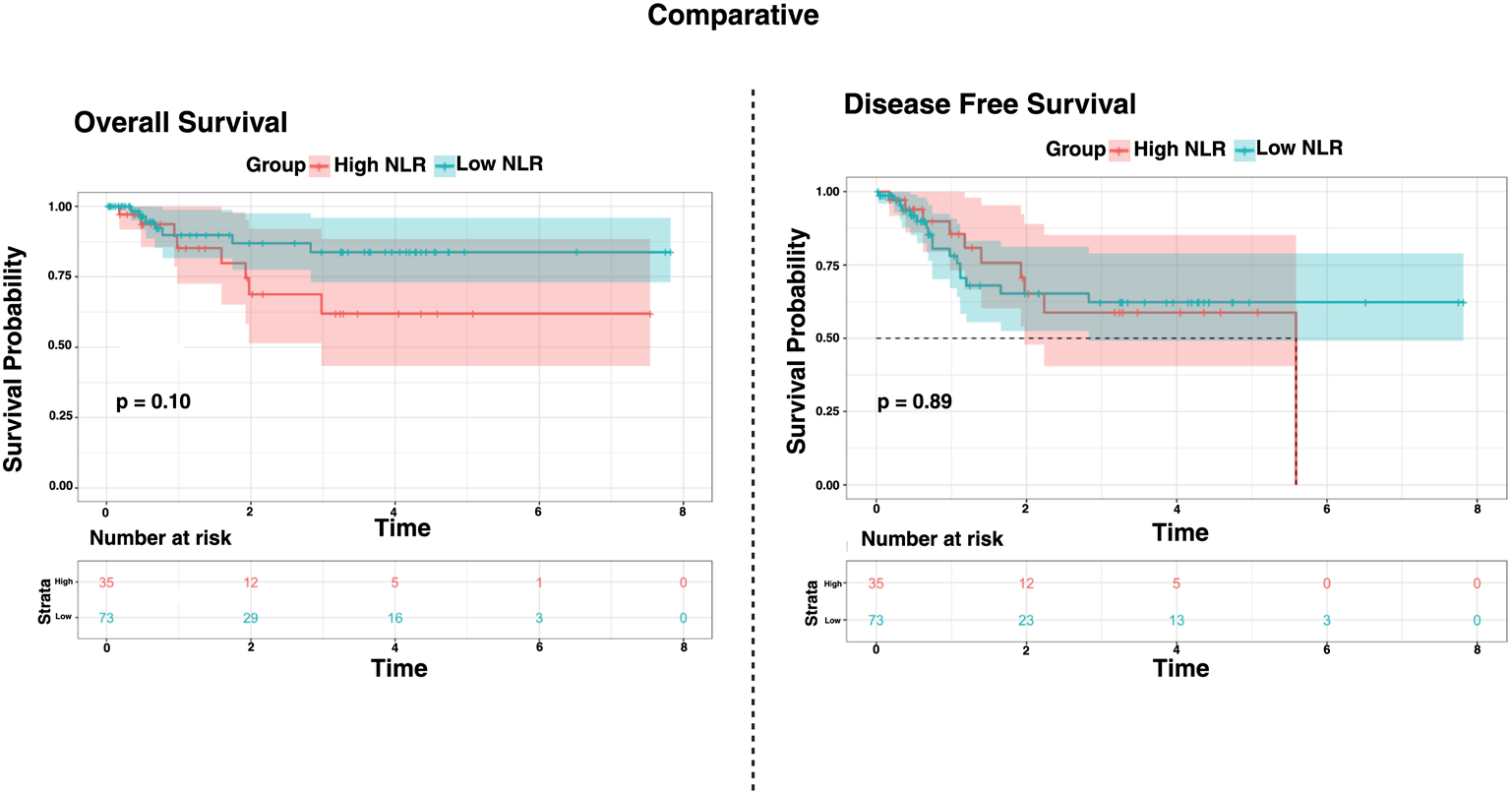
Kaplan–Meier curves demonstrate no statistically significant differences between high–NLR (H–NLR) and low–NLR (L–NLR) groups for either overall survival (left, *p* = 0.1) or disease–free survival (right, *p* = 0.89).

108 patients were included in the control cohort with the following characteristics: 63.7 years (SD = 10.8), 49 (45%) p16‐associated tumors, 51 (47%) tumors of the oropharynx, and an average pre‐treatment NLR of 4.51 B/L (SD = 5.75) (Table S1). 51% of patients underwent adjuvant radiotherapy, and 27% underwent adjuvant chemotherapy. 35 (32%) patients were classified as H‐NLR; 73 (68%) patients were classified as L‐NLR. Kaplan–Meier analysis of OS did not identify an improvement in OS (HR 0.44, 95% CI 0.16–1.22, *p* = 0.113) or DFS (HR 0.95, 95% CI 0.43–2.0, *p* = 0.895) in patients with L‐NLR compared to H‐NLR (Figure 3).

### 3.5 Pre‐ and Post‐Treatment Peripheral Cytokine Profiles

The concentrations (pg/mL) of circulating cytokines were compared between patients with H‐NLR and L‐NLR (Table 4). Across the entire patient population, patients with L‐NLR had significantly higher pre‐treatment concentrations of TGFα (*p* = 0.04) and post‐treatment concentrations of MCP3 (*p* = 0.049) and IL‐13 (*p* = 0.018) compared to patients with H‐NLR. Among patients who received Nivolumab + IDO inhibitor, patients with H‐NLR had significantly elevated concentrations of IL‐6 both pre‐ and post‐treatment compared to patients with L‐NLR (*p* = 0.04). In patients who received Nivolumab + Tadalafil, patients with L‐NLR had significantly higher pre‐treatment concentrations of EGF (*p* = 0.002). Among patients who received Nivolumab alone, TGFα (*p* = 0.005), IL‐17α (*p* = 0.041), IL‐13 (*p* = 0.041), IL‐1RA (*p* = 0.005), and RANTES (*p* = 0.030) were elevated pre‐treatment in patients with L‐NLR. TGFα (*p* = 0.009), IL‐17α (*p* = 0.043), IL‐1RA (*p* = 0.015), IL‐1b (*p* = 0.048), IL‐10 (*p* = 0.026), IL‐13 (*p* = 0.033), Fractakline (*p* = 0.041), and MPC3 (*p* = 0.039) were significantly higher post‐treatment in patients with L‐NLR compared to patients with H‐NLR. Among patients who received Durvalumab + Metformin, patients with L‐NLR demonstrated a greater decline in TNFα (*p* = 0.026) following ICI treatment from pre‐treatment levels compared to patients with H‐NLR. In patients who received Nivolumab monotherapy, patients with L‐NLR demonstrated a significant increase in IL‐15 following ICI therapy, whereas patients with H‐NLR showed a significant decline following treatment (*p* = 0.026).

**TABLE 4:** Differential Expression of Cytokines Stratified by Neutrophil‐to‐Lymphocyte Ratio (NLR).

Cytokine	Pre‐treatment H‐NLR/L‐NLR	*p*	Post‐treatment H‐NLR/L‐NLR	*p*
TGFα	**↓**	**0.04**	—	
RANTES	**↓**	**0.08**	—	
MCP3	—	—	**↓**	**0.049**
IL‐13	—	—	**↓**	**0.018**
IL‐6	**↑**	**0.04**	**↑**	**0.04**
EGF	**↓**	**0.002**	—	
CD40L	**↓**	0.090	—	
MCP1	**↓**	0.090	—	
MCP1	**↓**	0.090	—	
TGFα	**↓**	**0.005**	**↓**	**0.009**
TNFβ	**↓**	0.076	**↓**	0.09
IL‐17α	**↓**	**0.041**	**↓**	**0.043**
IL‐13	**↓**	0.041	**↓**	**0.033**
IL‐1RA	**↓**	**0.005**	**↓**	**0.015**
RANTES	**↓**	0.033	—	—
INFα	—	—	**↓**	0.083
INFy	—	—	**↓**	0.076
TNFβ	—	—	**↓**	0.091
IL‐1b	—	—	**↓**	**0.048**
IL‐10	—	—	**↓**	**0.026**
IL‐13	—	—	**↓**	**0.033**
Fractakline	—	—	**↓**	**0.041**
MPC3	—	—	**↓**	**0.039**

*Note:* Bold values indicate statistical significance (*p* < 0.05).

## 4 Discussion

NLR is an established prognostic biomarker as previous studies have demonstrated an association between high NLR and worse clinical outcomes across various solid tumor types [18, 19, 20]. This finding has been extended to HNSCC, where high pre‐treatment NLR is predictive of worse overall survival in patients who received treatment with chemoradiation or ICI for recurrent/metastatic disease [8, 13]. Our work is the first to assess the prognostic significance of this peripheral biomarker in HNSCC patients who received neoadjuvant ICI prior to definitive treatment. In our study, we identified an NLR cutoff of 4.14, above which OS and DFS significantly declined across the cohort. This finding corroborates and adds to the growing body of literature suggesting the prognostic utility of peripheral blood markers of inflammation that may aid in appropriate patient selection. Moreover, H‐NLR remained associated with poor survival among both responders and non‐responders, respectively. This novel relationship has not been evaluated previously among patients with HNSCC receiving ICI and encourages further investigation into the utility of pre‐treatment NLR as an independent prognostic factor. When applied to a control cohort of patients who were not treated with ICI, our pre‐treatment NLR cutoff did not demonstrate an association with survival, suggesting its specificity to nICI therapy.

Our results support previous studies that underscore the significance of circulating blood cells as indicators of inflammation within the local tumor microenvironment. NLR may serve as a surrogate marker of both inflammation and tumor progression, as neutrophils are capable of secreting immunosuppressive mediators and angiogenic factors, including reactive oxygen species, VEGF (vascular endothelial growth factor), and MMP‐9 (matrix metalloproteinase 9) possibly facilitating a tumor‐promoting microenvironment [18, 19]. Moreover, tumor‐associated neutrophils (TANs) have been shown to promote tumor growth, elevate immunosuppression in the tumor microenvironment, and decrease CD8+ cytotoxic T lymphocytes [20]. In contrast, low levels of circulating lymphocytes may correspond to fewer tumor‐infiltrating lymphocytes and a diminished anti‐tumor T‐cell response, further contributing to an immunosuppressive tumor microenvironment that can potentially lower the response to ICI [21]. Inflammation is a hallmark of cancer, and the reactivation of lymphocytes by ICIs is crucial for facilitating their anti‐tumor effects. Thus, relative neutrophil and lymphocyte levels reflect the tumor‐immune system interaction and can serve as key indicators in predicting the effectiveness of ICI therapy [22].

Our results demonstrated a variable peripheral cytokine profile based on pre‐treatment NLR. Previous work in the colorectal cancer population has corroborated these findings, with a demonstrated association between H‐NLR and expression of IL‐6, IL‐8, IL‐2Rα, all of which are related to key biological processes involved in carcinogenesis [23]. In our cohort of patients who received nivolumab, those with L‐NLR demonstrated a significant increase in IL‐15 levels while H‐NLR showed a decline following therapy. IL‐15 is a potentially potent immunomodulator through its ability to activate effector T cells and natural killer (NK) cells. A recent clinical trial of nivolumab in combination with an IL‐15 agonist in the management of NSCLC reported an improved anti‐tumor immune response. As such, it is possible that the decline in IL‐15 levels in our H‐NLR cohort reflects a poor immune profile that is less amenable to ICI therapy and may contribute to the observed patterns in survival.

Although our study demonstrated an association between pre‐treatment NLR and survival, we did not identify a significant association between NLR and PTE in patients treated with nICI, as previously demonstrated in ICI therapy [24]. The lack of association with PTE could be due to a combination of our small sample size and the challenges of achieving PTE using ICIs due to host‐ and tumor‐intrinsic functional heterogeneity that may facilitate an unfavorable environment for immune activation and tumor eradication [25, 26, 27]. Nevertheless, given that this is the first study to assess NLR in patients with HNSCC treated with nICI, further studies are needed to clarify these findings and explore their implications.

This study has several limitations, including those inherent to its retrospective, single‐institution nature. Despite including patients from three different clinical trials of neoadjuvant ICI, the sample size is relatively small and comprised largely of white males, limiting the power of our analysis and the generalizability of our findings. Furthermore, our trials had strict exclusion criteria for patients with a history of previous radiotherapy, chemotherapy, prior malignancies, and hematological disorders, which could potentially influence the NLR values of our population. NLR can also be affected by stress triggers that might precipitate an elevated neutrophil count but were not assessed in this study, such as febrile neutrophilia. Additionally, we did not further characterize neutrophils by their phenotypes; thus, the heterogeneity of pro‐tumorigenic and anti‐tumorigenic neutrophil phenotypes could not be evaluated. Future studies should pursue a multi‐institutional prospective design with a larger sample size to allow for greater generalizability, more extensive validation, and more concrete associations between NLR and ICI response and survival. Moreover, the specific cutoff of 4.14 found in this study encourages further investigation to determine the optimal NLR cutoff value to facilitate improved patient selection and counseling.

## 5 Conclusion

Our study suggests that high pre‐treatment NLR was predictive of poor prognosis among patients with resectable HNSCC undergoing neoadjuvant ICI. This association persisted upon sub‐stratification for p16 status, ICI response status, and ICI trial. Routine, cost‐effective peripheral biomarkers such as NLR demonstrate potential for improving patient selection. Further studies are needed to validate the NLR as a biomarker within this patient population to inform clinical use.

## Author Contributions

**Pablo Llerena:** conceptualization (equal), formal analysis (equal), investigation (equal), writing – original draft (equal), writing – review and editing (equal). **Praneet C. Kaki:** data curation (equal), writing – original draft (equal), writing – review and editing (equal). **Kathryn Nunes:** writing – original draft (equal), writing – review and editing (equal). **Eric Mastrolonardo:** methodology (equal), writing – original draft (equal), writing – review and editing (equal). **Kelly Bridgham:** writing – original draft (equal), writing – review and editing (equal). **Annie E. Moroco:** writing – original draft (equal), writing – review and editing (equal). **Hani Samarah:** writing – original draft (equal), writing – review and editing (equal). **Mouadh Barbirou:** data curation (equal), formal analysis (equal). **David Baek:** writing – review and editing (equal). **Chun Wang:** data curation (equal), formal analysis (equal). **Voichita Bar Ad:** writing – original draft (equal), writing – review and editing (equal). **David M. Cognetti:** project administration (equal), writing – review and editing (equal). **Joseph M. Curry:** project administration (equal), writing – review and editing (equal). **Jennifer M. Johnson:** formal analysis (equal), project administration (equal), writing – review and editing (equal). **Hushan Yang:** data curation (equal), writing – review and editing (equal). **Larry A. Harshyne:** investigation (equal), project administration (equal), writing – review and editing (equal). **Adam J. Luginbuhl:** conceptualization (equal), formal analysis (equal), investigation (equal), methodology (equal), project administration (equal), writing – original draft (equal), writing – review and editing (equal).

## Funding

The authors have nothing to report.

## Conflicts of Interest

The authors declare no conflicts of interest.

## Supporting information


**Data S1:** cam471693‐sup‐0001‐Supinfo.docx.

## Data Availability

The data that support the findings of this study are available from the corresponding author upon reasonable request.
