# Supplementary material for: Neutrophil‐to‐Lymphocyte Ratio as a Prognostic Factor of Survival Outcomes in Head and Neck Squamous Cell Carcinoma Receiving Neoadjuvant Immunotherapy
Source: Cancer Med. 2026 Mar 22;15(3):e71693. doi: 10.1002/cam4.71693 (PMC13140833; doi:10.1002/cam4.71693)
Supplement: Supplementary file 1 — Data S1: cam471693‐sup‐0001‐Supinfo.docx. [file CAM4-15-e71693-s001.docx]

Supplementary Tables & Figures

**sTable 1: Patient and Clinicopathologic Characteristics by ICI trials**

| **Characteristic** | **N** | **Overall**, N = 97*^1^* | **Durvalumab +/- Metformin**, N = 31*^1^* | **Nivolumab +/- IDO**, N = 36*^1^* | **Nivolumab +/- Tadalafil**, N = 30*^1^* | **p-value***^2^* |
| --- | --- | --- | --- | --- | --- | --- |
| Age (years) | 97 | 61.59 (10.62) | 59.55 (11.29) | 62.72 (10.34) | 62.33 (10.30) | 0.6 |
| Sex | 97 |  |  |  |  | 0.12 |
| Female |  | 16 (16%) | 8 (26%) | 6 (17%) | 2 (6.7%) |  |
| Male |  | 81 (84%) | 23 (74%) | 30 (83%) | 28 (93%) |  |
| Race | 97 |  |  |  |  | 0.5 |
| Black or African American |  | 6 (6.2%) | 2 (6.5%) | 1 (2.8%) | 3 (10%) |  |
| Other Pacific Islander |  | 1 (1.0%) | 1 (3.2%) | 0 (0%) | 0 (0%) |  |
| White |  | 90 (93%) | 28 (90%) | 35 (97%) | 27 (90%) |  |
| Baseline BMI (kg/m2) | 97 | 28.86 (6.91) | 29.26 (8.73) | 28.46 (5.98) | 28.92 (5.94) | >0.9 |
| Smoking Status | 97 |  |  |  |  | 0.2 |
| Current |  | 18 (19%) | 3 (9.7%) | 10 (28%) | 5 (17%) |  |
| Former |  | 40 (41%) | 13 (42%) | 11 (31%) | 16 (53%) |  |
| Never |  | 39 (40%) | 15 (48%) | 15 (42%) | 9 (30%) |  |
| p16+ HPV-related disease | 97 | 56 (58%) | 21 (68%) | 17 (47%) | 18 (60%) | 0.2 |
| Pathologic AJCC Stage | 96 |  |  |  |  | 0.4 |
| 0 |  | 5 (5.2%) | 0 (0%) | 3 (8.3%) | 2 (6.9%) |  |
| I |  | 48 (50%) | 18 (58%) | 16 (44%) | 14 (48%) |  |
| II |  | 11 (11%) | 3 (9.7%) | 3 (8.3%) | 5 (17%) |  |
| III |  | 6 (6.2%) | 2 (6.5%) | 1 (2.8%) | 3 (10%) |  |
| IVa |  | 20 (21%) | 5 (16%) | 10 (28%) | 5 (17%) |  |
| IVb |  | 6 (6.2%) | 3 (9.7%) | 3 (8.3%) | 0 (0%) |  |
| T4b |  | 1 (1.0%) | 1 (3.2%) | 0 (0%) | 0 (0%) |  |
| Tis |  | 1 (1.0%) | 0 (0%) | 1 (2.8%) | 0 (0%) |  |
| TX |  | 1 (1.0%) | 0 (0%) | 0 (0%) | 1 (3.4%) |  |
| Adjuvant Therapy | 97 |  |  |  |  | 0.11 |
| Chemoradiotherapy |  | 16 (16%) | 3 (9.7%) | 6 (17%) | 7 (23%) |  |
| Chemotherapy |  | 1 (1.0%) | 1 (3.2%) | 0 (0%) | 0 (0%) |  |
| None |  | 32 (33%) | 6 (19%) | 15 (42%) | 11 (37%) |  |
| Radiotherapy |  | 48 (49%) | 21 (68%) | 15 (42%) | 12 (40%) |  |
| Follow-up Duration (Months) | 97 | 34.32 (15.94) | 34.26 (12.35) | 23.89 (9.93) | 46.90 (16.31) | <0.001 |
| *^1^*Mean (SD); n (%)  *^2^*Kruskal-Wallis rank sum test; Fisher's exact test; Pearson's Chi-squared test | | | | | | |

**Table s2** Significant Cytokine analysis across all trials Post vs Pre comparison

(p <0.05)

| Trial | Cyotkine | H-NLR Change (Post-Pre) | L-NLR Change  (Post-Pre) | P value |
| --- | --- | --- | --- | --- |
| **All trials** | - | **-** | **-** | - |
| **Durva Met** | **TNFα** | **+11.33 (5.97)** | -24.04 (49.41) | **0.026** |
| Durva Alone | - | - | - | - |
| NivoIDO | - | - | - | - |
| NivoTad | - | **-** | **-** | - |
| Nivoalone | IL-15 | -11.19 (11.17) | +6.19 (16.13) |  |


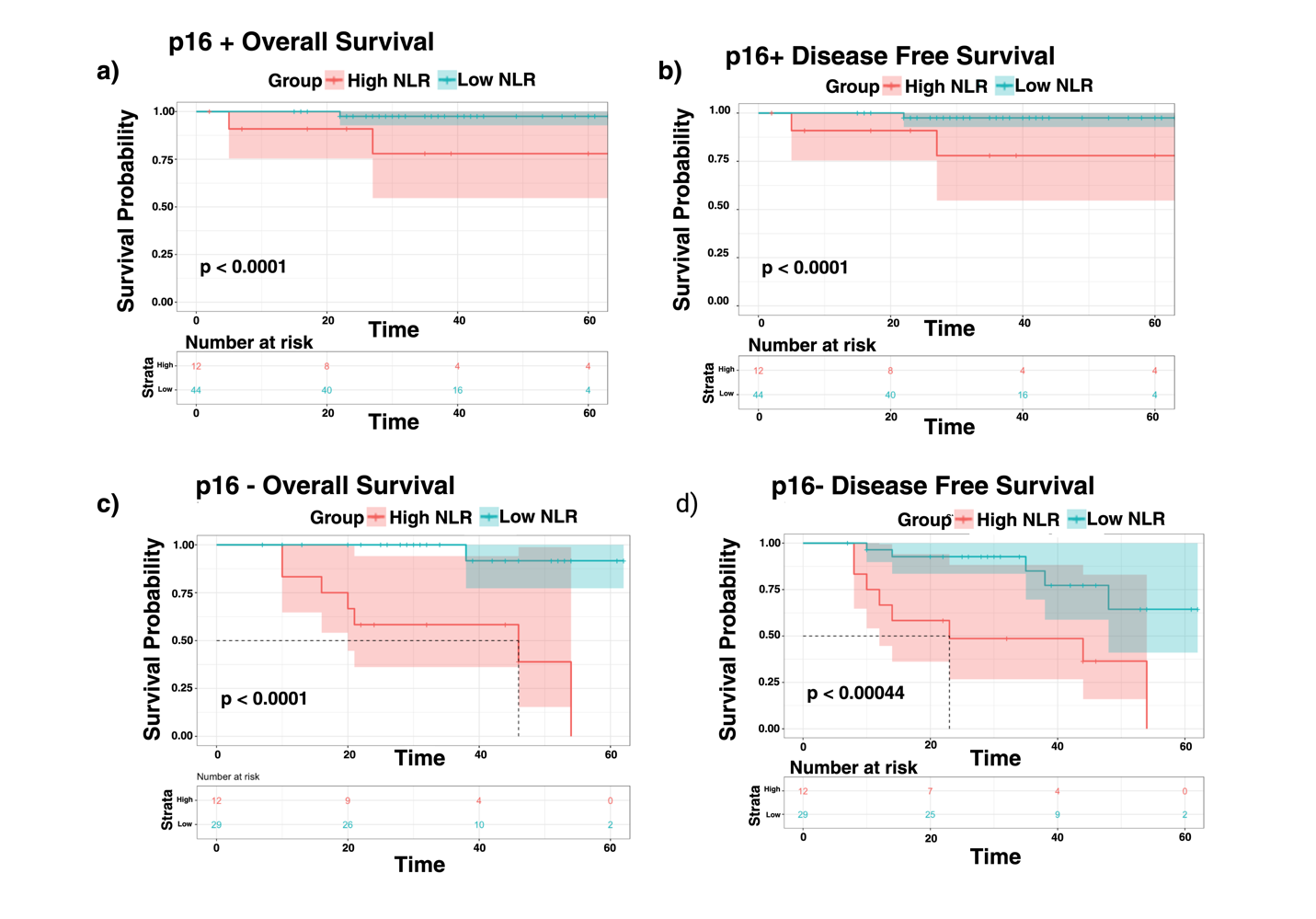


**Figure S1:** Kaplan-Meier Survival Analysis Stratified by p16 Status and NLR in HNSCC Patients. **(a) p16+ Overall Survival:** Significant difference in OS between high NLR (H-NLR) and low NLR (L-NLR) groups (p < 0.0001). **(b) p16+ Disease-Free Survival:** Significant difference in DFS between H-NLR and L-NLR groups (p < 0.0001). **(c) p16- Overall Survival:** Significant difference in OS between H-NLR and L-NLR groups (p < 0.0001). **(d) p16- Disease-Free Survival:** Significant difference in DFS between H-NLR and L-NLR groups (p < 0.00044).


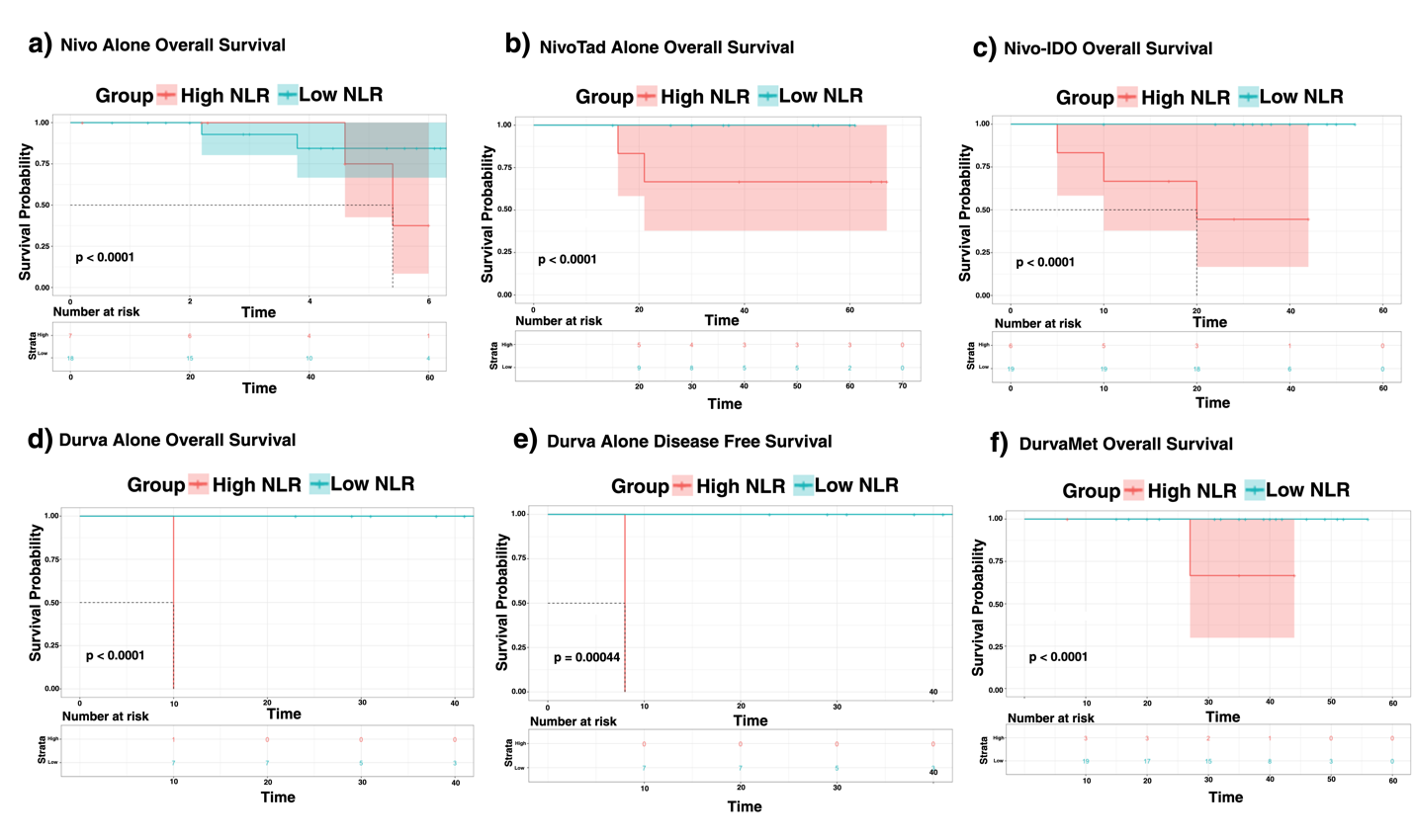


**Figure S2:** Kaplan-Meier Survival Analysis by NLR Stratified by Treatment Regimen in HNSCC Patients. (**a) Nivolumab Alone Overall Survival:** Significant difference in OS between high NLR (H-NLR) and low NLR (L-NLR) groups (p < 0.0001). **(b) Nivolumab + Tadalafil (NivoTad) Overall Survival:** Significant difference in OS between H-NLR and L-NLR groups (p < 0.0001). **(c) Nivolumab + BMS986205 (Nivo-IDO) Overall Survival:** Significant difference in OS between H-NLR and L-NLR groups (p < 0.0001). **(d) Durvalumab Alone Overall Survival:** Significant difference in OS between H-NLR and L-NLR groups (p < 0.0001). **(e) Durvalumab Alone Disease-Free Survival:** Significant difference in DFS between H-NLR and L-NLR groups (p = 0.00044). **(f) Durvalumab + Metformin (DurvaMet) Overall Survival:** Significant difference in OS between H-NLR and L-NLR groups (p < 0.0001).


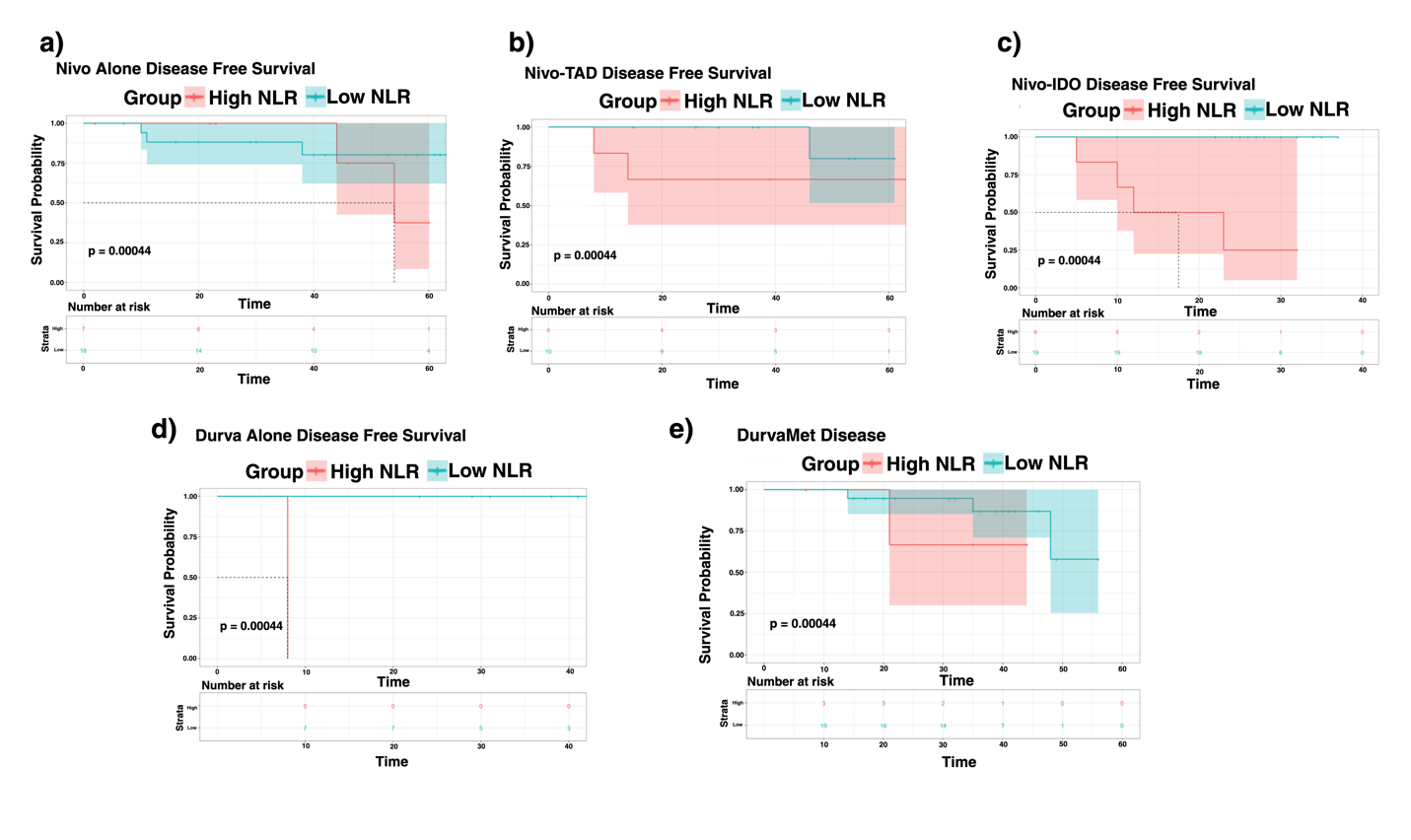
**Figure S3:** Disease-Free Survival Analysis by NLR Stratified by Treatment Regimen in HNSCC Patients. **(a) Nivolumab Alone Disease-Free Survival:** Significant difference in DFS between high NLR (H-NLR) and low NLR (L-NLR) groups (p = 0.00044). **(b) Nivolumab + Tadalafil (Nivo-TAD) Disease-Free Survival:** Significant difference in DFS between H-NLR and L-NLR groups (p = 0.00044). **(c) Nivolumab + BMS986205 (Nivo-IDO) Disease-Free Survival:** Significant difference in DFS between H-NLR and L-NLR groups (p = 0.00044). **(d) Durvalumab Alone Disease-Free Survival:** Significant difference in DFS between H-NLR and L-NLR groups (p = 0.00044). **(e) Durvalumab + Metformin (DurvaMet) Disease-Free Survival:** Significant difference in DFS between H-NLR and L-NLR groups (p = 0.00044)

**Supplementary Methods**

#### Window of opportunity trial of nivolumab and tadalafil in patients with squamous cell carcinoma of the head and neck (NCT03238365) (Nivo±Tad)

This was an investigator-initiated, two-arm multi-institutional (Thomas Jefferson University and Vanderbilt University) randomized trial involving patients with newly diagnosed and resectable HNSCC of any stage by the American Joint Committee on Cancer Criteria (AJCC) 8th edition. Please review Luginbuhl et al for methodology of the trial.

*Window of opportunity trial of nivolumab and BMS986205 in patients with squamous cell carcinoma of the head and neck (NCT03854032) (Nivo±IDO)*

This was a phase I investigator-initiated, two-arm randomized trial involving patients with any-stage (AJCC 8th edition) resectable HNSCC. Participants were randomized in a 3:1 ratio to receive either nivolumab combined with BMS986205 (an Indoleamine 2,3-dioxygenase [IDO] inhibitor) or nivolumab alone. Patients in the nivolumab + BMS986205 group received 100 mg of BMS986205 orally each day for 28 days, starting on day 3 (±3). Both groups received a single dose of nivolumab, 480 mg IV, on day 10 (±3). Tumor radiographic response at the primary tumor site and regional lymph nodes was assessed at approximately 5 weeks. Non-responders underwent definitive surgical resection at this time, while responders repeated their treatment for an additional 4 weeks according to their initial randomization arm, followed by definitive surgical resection.

#### Window of opportunity for durvalumab (MEDI4736) plus metformin trial in squamous cell carcinoma of the head and neck (NCT03618654) (Durva±Met)

#### This was a phase I investigator-initiated, two-arm randomized trial. Patients with any-stage (AJCC 8th edition) resectable HNSCC were included. Subjects were randomized 3:1 to the programmed death-ligand 1 (PD-L1) inhibitor durvalumab + metformin or durvalumab alone. Patients in the durvalumab + metformin arm received a four-week supply of metformin to begin on day 1 (±2). Subjects in this arm began with metformin 500 mg oral daily for 3 days, titrated to 500 mg twice daily for an additional 3 days. If tolerated, the dose was again increased to 1000 mg twice daily after day 6. Patients maintained the maximum tolerated dose until the day prior to surgery. All subjects received 1500 mg durvalumab (MEDI4736) via IV infusion on day 3 (±2). Definitive surgical resection was performed for approximately 4 weeks.

*Pathological Treatment Effect: Responder and Non responder status*

Treatment response, defined as a pathologic treatment effect (pTE) greater than 20%, was evaluated as a secondary endpoint. All trials included definitive cancer resection. Based on the pTE of the post-treatment specimen, patients were classified as responders or non-responders. In the Nivo±Tad and Nivo±IDO trials, responders were defined as having a pTE% ≥ 20%, while non-responders had a pTE% of < 20%, based on the average pTE at the primary site and lymph nodes. (Durva±Met) responders were defined as having a pTE% ≥ 10%, while non-responders had a pTE% of < 10%,

**Supplemental materials**

#### Window of opportunity trial of nivolumab and tadalafil in patients with squamous cell carcinoma of the head and neck (NCT03238365) (Nivo±Tad)

This was an investigator-initiated, two-arm multi-institutional (Thomas Jefferson University and Vanderbilt University) randomized trial involving patients with newly diagnosed and resectable HNSCC of any stage by the American Joint Committee on Cancer Criteria (AJCC) 8th edition. Please review Luginbuhl et al for methodology of the trial.

*Window of opportunity trial of nivolumab and BMS986205 in patients with squamous cell carcinoma of the head and neck (NCT03854032) (Nivo±IDO)*

This was a phase I investigator-initiated, two-arm randomized trial involving patients with any-stage (AJCC 8th edition) resectable HNSCC. Participants were randomized in a 3:1 ratio to receive either nivolumab combined with BMS986205 (an Indoleamine 2,3-dioxygenase [IDO] inhibitor) or nivolumab alone. Patients in the nivolumab + BMS986205 group received 100 mg of BMS986205 orally each day for 28 days, starting on day 3 (±3). Both groups received a single dose of nivolumab, 480 mg IV, on day 10 (±3). Tumor radiographic response at the primary tumor site and regional lymph nodes was assessed at approximately 5 weeks. Non-responders underwent definitive surgical resection, while responders repeated their treatment for an additional 4 weeks according to their initial randomization arm, followed by definitive surgical resection.

#### Window of opportunity for durvalumab (MEDI4736) plus metformin trial in squamous cell carcinoma of the head and neck (NCT03618654) (Durva±Met)

#### This was a phase I investigator-initiated, two-arm randomized trial. Patients with any-stage (AJCC 8th edition) resectable HNSCC were included. Subjects were randomized 3:1 to the programmed death-ligand 1 (PD-L1) inhibitor durvalumab + metformin or durvalumab alone. Patients in the durvalumab + metformin arm received a four-week supply of metformin to begin on day 1 (±2). Subjects in this arm began with metformin 500 mg oral daily for 3 days, titrated to 500 mg twice daily for an additional 3 days. If tolerated, the dose was again increased to 1000 mg twice daily after day 6. Patients maintained the maximum tolerated dose until the day prior to surgery. All subjects received 1500 mg durvalumab (MEDI4736) via IV infusion on day 3 (±2). Definitive surgical resection was performed for approximately 4 weeks.

*Pathological Treatment Effect: Responder and Non responder status*

Treatment response, defined as a pathologic treatment effect (pTE) greater than 20%, was evaluated as a secondary endpoint. All trials included definitive cancer resection. Based on the pTE of the post-treatment specimen, patients were classified as responders or non-responders. In the Nivo±Tad and Nivo±IDO trials, responders were defined as having a pTE% ≥ 20%, while non-responders had a pTE% of < 20%, based on the average pTE at the primary site and lymph nodes.
